# Factors Shaping Trust and Satisfaction With AI Medical Chatbots: A Mixed Methods Vignette Survey of Caregivers Seeking Guidance on Pediatric Infectious Diseases

**DOI:** 10.2196/88126

**Published:** 2026-07-14

**Authors:** Run Huang, Joseph Cecil, Marjorie Freedman, Souti Chattopadhyay

**Affiliations:** 1 Thomas Lord Department of Computer Science, University of Southern California, 941 Bloom Walk, Los Angeles, CA, 90089, United States, 1 2137404530; 2 Information Sciences Institute, University of Southern California, Los Angeles, CA, United States

**Keywords:** medical chatbot, artificial intelligence in health care, large language model, artificial intelligence, ChatGPT, chatbot

## Abstract

**Background:**

As artificial intelligence (AI) chatbots become an increasingly common source of quick medical guidance, it is important to understand whether their responses meet users’ needs and support well-informed health decisions. Yet, existing evaluation frameworks rely primarily on expert-defined evaluation dimensions that have not been empirically validated with end users. It remains unclear whether these frameworks capture the criteria people actually use when judging a response to be useful, trustworthy, and satisfying, or which evaluation dimensions matter most to users in practice.

**Objective:**

This study empirically examined how commonly used dimensions such as Accuracy and Comprehensiveness shape caregivers’ satisfaction with AI-generated answers to pediatric health questions. We further investigated what expectations and communication needs may be overlooked by current evaluation frameworks.

**Methods:**

We conducted a mixed methods vignette survey with 191 caregivers recruited through Prolific. Participants evaluated GPT-4o responses to a set of clinician-approved pediatric health questions across 8 dimensions and rated overall satisfaction. We quantified the influence of each dimension on overall satisfaction using a Cumulative Link Mixed Model and performed an inductive thematic analysis of open-ended comments to identify gaps in established frameworks.

**Results:**

A total of 191 caregivers evaluated 1146 chatbot responses. Initially, caregivers rated Accuracy and Credibility as the most important dimensions. However, Cumulative Link Mixed Model analysis identified Usefulness as the strongest driver of overall satisfaction (odds ratio [OR] 2.53, 95% CI 1.95‐3.27; *P*<.001), followed by Thoroughness (OR 2.15, 95% CI 1.69‐2.73; *P*<.001). Comprehensiveness did not significantly influence satisfaction (OR 1.05, 95% CI 0.82‐1.33; *P*=.71). Qualitative feedback helped explain this: participants frequently criticized responses as “too long” and preferred concise, actionable guidance. Empathy/Warmth was significantly associated with overall satisfaction (OR 1.48, 95% CI 1.27‐1.72; *P*<.001) but elicited polarized reactions (Van der Eijk agreement μ=0.38): some caregivers valued emotional support, while others found AI-generated empathy insincere and undermined credibility. Medical disclaimers increased trust in higher-risk situations but reduced confidence in lower-risk scenarios.

**Conclusions:**

Existing evaluation frameworks only partially capture how caregivers assess medical chatbots. Caregivers valued actionable guidance, credible references, and clear reasoning over lengthy, exhaustive detail. Rather than passively receiving dense information, they preferred an interactive style in which the chatbot proactively proposed follow-up suggestions, helping them steer the conversation toward their specific needs. Reactions to empathetic language and medical disclaimers were context-dependent: features that built trust in some situations could seem insincere, excessive, or unnecessary in others. These findings suggest that future medical chatbots should move beyond one-size-fits-all communication and adapt to individual users’ situations, preferences, and information needs. Evaluation protocols should likewise assess not only whether chatbot responses are accurate and comprehensive but also whether they are actionable, appropriately toned, and responsive to users’ evolving needs over the course of a conversation.

## Introduction

Artificial intelligence (AI) chatbots powered by large language models are transforming how people seek medical advice [1-5]. Rather than sifting through various sources or waiting for a clinic appointment, people can now get instant answers from general-purpose chatbots such as ChatGPT and Claude [6-8]. This convenience has attracted millions of users, particularly among caregivers seeking immediate guidance when a child develops early symptoms [9-15]. Even people who never intentionally seek medical advice from a chatbot may still encounter it through AI-generated summaries placed at the top of search results on Google and Bing.

However, such ubiquity also raises the stakes for quality [16-19]. A response that is inaccurate, unclear, or off tone could delay necessary care [20], trigger undue anxiety [21], or even reinforce medical misinformation [22]. As such, ensuring that patient-facing chatbots deliver responses that are not only factually correct but also comprehensible, actionable, and trustworthy to lay users is not merely a technical challenge but also a public health priority [20,23,24].

Existing evaluation methods for medical chatbots fall into 2 categories: automatic metrics and human evaluation frameworks [25-27]. Automatic approaches include reference-based factuality metrics that measure whether a response aligns with gold-standard answers, as well as general natural language generation metrics such as readability scores, fluency measures, and semantic similarity. While these methods are scalable and reproducible, they capture only surface-level properties of text [28-30]. They say little about the qualities that actually shape whether a user feels comfortable acting on a chatbot’s advice, such as whether the response is easy to understand, appropriately scoped, or conveys an appropriate tone. As a result, computational metrics alone capture only a narrow slice of what determines response quality in practice [28,29].

To this end, several human evaluation frameworks have been proposed. Degachi et al [28] and Singhal et al [31] developed expert evaluation frameworks through interviews with a dozen medical professionals. Tam et al [29], Abbasian et al [32], and Sallam et al [33] synthesized common evaluation dimensions from prior literature. While these contributions go a long way toward standardizing the process of human evaluation for medical chatbots, they are primarily derived top-down from theoretical models and literature reviews, without empirical evidence from the end users to support their validity and effectiveness. Therefore, it remains unclear which dimensions genuinely drive user satisfaction and whether users interpret them consistently.

This gap has real practical consequences. Evaluation frameworks built primarily from expert perspectives may prioritize dimensions that have little bearing on user experience, while overlooking considerations that emerge only through real interactions with chatbots [28]. For instance, as our findings will show, friendly, empathetic language can sometimes undermine rather than build trust, depending on the clinical context. Such nuances are difficult to anticipate from theory alone and underscore the need for empirical, user-centered validation of evaluation frameworks.

To address this gap, we conducted an exploratory study to examine how established evaluation dimensions influence user satisfaction in practice and to identify nuances that existing frameworks may miss. We focused on caregivers seeking advice about common pediatric infectious diseases. This population is well suited for 3 reasons. First, prior work has relied heavily on clinicians’ perspectives as proxies for patient needs [28,31], leaving caregivers’ own evaluation criteria largely underrepresented. Second, caregivers are a primary group of end users of patient-facing medical chatbots, often seeking quick, lay-friendly guidance on how to respond to a child’s symptoms [8]. Third, pediatric infections are common, acute conditions that can carry longer-term health implications [34], for which caregivers usually turn to online resources before seeking professional care [35], making them a realistic and representative scenario for chatbot use.

Our study seeks to answer the following research questions (RQs):

- RQ1: How do caregivers rate the importance of established evaluation dimensions?
- RQ2: How satisfied are caregivers with chatbot responses across these dimensions?
- RQ3: How does each dimension influence caregivers’ overall satisfaction?
- RQ4: What evaluation considerations emerge beyond existing frameworks?

To answer these questions, we conducted a mixed methods vignette survey with 191 caregivers across the United States, combining quantitative modeling with inductive thematic analysis. To our knowledge, this is the first study to quantitatively model how established evaluation dimensions contribute to caregivers’ overall satisfaction with medical chatbot responses. We translate these findings into concrete design implications for refining human evaluation protocols and guiding the design of future patient-facing medical chatbots.

## Methods

### Overview

We conducted a mixed methods vignette survey to examine how caregivers evaluated AI chatbot responses to pediatric health questions. In this study, a vignette refers to 1 caregiver question presented with 2 alternative chatbot responses in different communication styles, such as brief or formal. Each participant evaluated 3 vignettes (ie, 6 chatbot responses), allowing them to compare responses that varied in tone, length, and information density. For each response, participants rated it on 8 commonly cited evaluation dimensions and their overall satisfaction. We then used a Cumulative Link Mixed Model (CLMM) to examine how ratings on these dimensions were associated with overall satisfaction, while accounting for repeated observations within participants and variation across questions and responses. We also conducted an inductive thematic analysis of participants’ open-ended comments to identify user concerns and potential gaps in existing evaluation frameworks.

### Ethical Considerations

This study was reviewed by the University of Southern California Institutional Review Board and was determined to be exempt human subjects research under Study ID UP-25-00074, “Understanding Interactions With Chatbots for Online Medical Information.” All participants were adults who self-reported being the primary caregiver of at least 1 child younger than 18 years. No children were recruited or enrolled as participants. The requirement for informed consent was waived as part of the exempt review determination. Participants were provided with study information before beginning the survey and were informed that participation was voluntary and that they could withdraw from the study at any time. Although survey questions generally required responses to proceed, participants could discontinue participation at any point. To protect participant privacy, survey responses were deidentified before analysis. The study did not collect participant names, contact information, child names, or directly identifiable medical information. Prolific identifiers were used only for participant management, compensation, and quality control purposes, and were converted to study-specific participant IDs before analysis. Access to the data was limited to research team members listed on the approved institutional review board protocol. Results are reported in aggregate, and open-ended responses are presented only as deidentified quotations. Participants were compensated through Prolific. Each participant received at least US $5 for completing the survey, with compensation adjusted when necessary based on actual completion time to meet or exceed Prolific’s minimum rate of US $9 per hour.

### Recruitment

We recruited participants across the United States through Prolific, a widely used platform in academic research that allowed us to reach a diverse pool of caregivers with varied ages, educational levels, and professional backgrounds [36,37]. Participants were required to be the primary caregiver of at least 1 child younger than 18 years, have prior experience seeking medical information online, and be a US resident fluent in either English or Spanish. Eligibility was confirmed through an additional screening survey. The survey was first developed in English and then translated into Spanish using GPT-4o. A native Spanish-speaking medical professional at Children’s Hospital Los Angeles reviewed the translation for clarity and medical appropriateness. We also used Gemini 2.0 to back-translate the Spanish version into English as an additional consistency check.

We focused on caregivers seeking advice about common pediatric infectious diseases because conditions such as the flu and ear infections are widespread, making it feasible to recruit participants with relevant firsthand experience. These illnesses also represent a typical chatbot use case: caregivers need quick, reliable guidance to assess symptom severity and decide whether to seek professional care, often under time pressure. This population is therefore both practically accessible and well suited for examining how lay caregivers perceive AI-generated medical guidance in situations that may require timely decisions.

### Survey Instrument

#### Evaluation Dimensions

We reviewed prior literature on human evaluation frameworks for medical AI chatbots and extracted more than 20 distinct dimensions [6,9,19,23,27-29,31,38-45]. Including all of them was impractical because asking respondents to rate 20+ dimensions would substantially increase the burden and risk compromising data quality. We therefore aimed to identify a parsimonious set that conceptually represented the breadth of dimensions proposed in prior work while remaining feasible to assess in a single survey session.

We narrowed the set of dimensions in 3 stages. First, we removed dimensions that require medical expertise to judge, such as whether the information aligns with current clinical guidelines or how harmful a response might be. Second, we removed dimensions that cannot be assessed from a single session, such as consistency across similar queries. Third, we conducted a pilot study with 40 caregivers to screen the remaining dimensions for redundancy. We calculated the variance inflation factor (VIF) for each dimension, which flagged several with scores above 5, suggesting multicollinearity. We then examined pairwise correlations among the high-VIF dimensions and found that strongly correlated pairs also overlapped conceptually in their definitions. For example, Trust and Credibility both reflected whether a response appears believable, while Style/Formatting and Readability both concerned presentation and structure. We therefore merged them into a single dimension, yielding a final set of 8. All VIF values among these 8 dimensions were below 5, indicating sufficient independence for regression modeling. Table 1 presents the 8 dimensions and their definitions.

**Table 1.** Evaluation dimensions distilled from existing frameworks.

Dimension	Definition
Accuracy	Perceived correctness of the response.
Clarity	The ease of understanding the medical content and reasoning in the response.
Comprehensiveness	The breadth of aspects covered in a single response.
Credibility	How believable and trustworthy the response appears.
Empathy/warmth	The level of empathy and care shown in the response.
Readability/style	The presentation of the response, including its tone, structure, and formatting.
Thoroughness	The level of detail and depth provided in a single response.
Usefulness	The degree to which the response provided is useful in answering the question, for example, guiding next steps, suggesting actions, indicating urgency, and so forth.

#### Vignettes and Stimulus

We compiled 40 representative caregiver questions by drawing on topics identified in prior studies. These questions were vetted by medical experts at the Children’s Hospital Los Angeles to ensure clinical relevance, realism, and appropriateness for caregiver-facing scenarios. Questions were revised based on their feedback.

To generate variation in how information was presented, we prompted GPT-4o to respond to each question in four communication styles identified in prior research [46-49]: (1) brief, catering to preferences for concise information in time-sensitive situations; (2) formal, reflecting traditional medical communication patterns; (3) evidence-based, incorporating citations and clinical references; and (4) empathetic, foregrounding emotional acknowledgment of caregivers’ concerns. This produced 4 responses per question that varied in tone, length, and information density, allowing participants to evaluate responses across a broader range of realistic chatbot behaviors rather than a single uniform response style.

#### Survey Design

The survey comprised 2 sections. The first captured participants’ prior expectations about chatbot responses, while the second elicited their evaluations of actual responses across the 8 dimensions.

##### Importance Ratings

Before viewing any chatbot responses, participants rated the importance of each of the 8 dimensions on a 5-point scale from “very low importance” to “very high importance.” To reduce ceiling effects, participants could assign no more than 3 dimensions to any single importance level, encouraging differentiation among dimensions. This step captured participants’ prior expectations about what makes a satisfying medical chatbot response, before they were exposed to actual chatbot output.

##### Vignette Evaluation

Each participant saw 3 randomly assigned vignettes. Each vignette consisted of 1 question paired with 2 response variants drawn from the 4 communication styles. For each response, participants rated all 8 dimensions and their overall satisfaction on a 5-point scale and provided open-ended comments describing what they liked and disliked. This comparative design allowed participants to make relative judgments, which tend to yield more reliable data than absolute ratings in isolation [50,51]. The open-ended comments revealed the reasoning behind respondents’ quantitative ratings and surfaced considerations not captured by the predefined dimensions.

### Statistical Analysis

#### Model

We used a CLMM to examine the driving factors of caregivers’ overall satisfaction. The model was fitted using the *ordinal* package in R (R Core Team) [52]. CLMM was chosen over alternative frameworks because it respects the ordinal nature of the outcome variable (measured on a 5-point Likert scale), while accounting for random effects. Given our repeated-measures design, we included three sources of random variation: (1) participant-level variability, to model individual differences in rating tendencies; (2) question-level variability, to adjust for differences across the 40 caregiver questions in topic, complexity, or typicality; and (3) response-level variability, to account for inherent differences across the 160 responses (40 questions×4 communication styles) in how they were perceived across participants. The final model specification was as follows:

Overall_satisfaction ~ Accuracy + Clarity + Comprehensiveness + Credibility + Empathy_Warmth + Readability_Style + Thoroughness + Usefulness + (1 | participant_id) + (1 | question_id) + (1 | response_id)

For each response, participants rated how satisfied they were with the response on each of the 8 evaluation dimensions. These ratings, coded as integers from 1 (dissatisfied) to 5 (satisfied), served as numeric predictors for the ordinal overall satisfaction outcome. We applied the Holm correction [53] to all reported *P* values to control for multiple comparisons across the 8 predictors. VIF scores for all predictors fell below the conventional threshold of 5, suggesting that multicollinearity was unlikely to compromise model estimation.

#### Model Diagnostics

**Figure 1.**
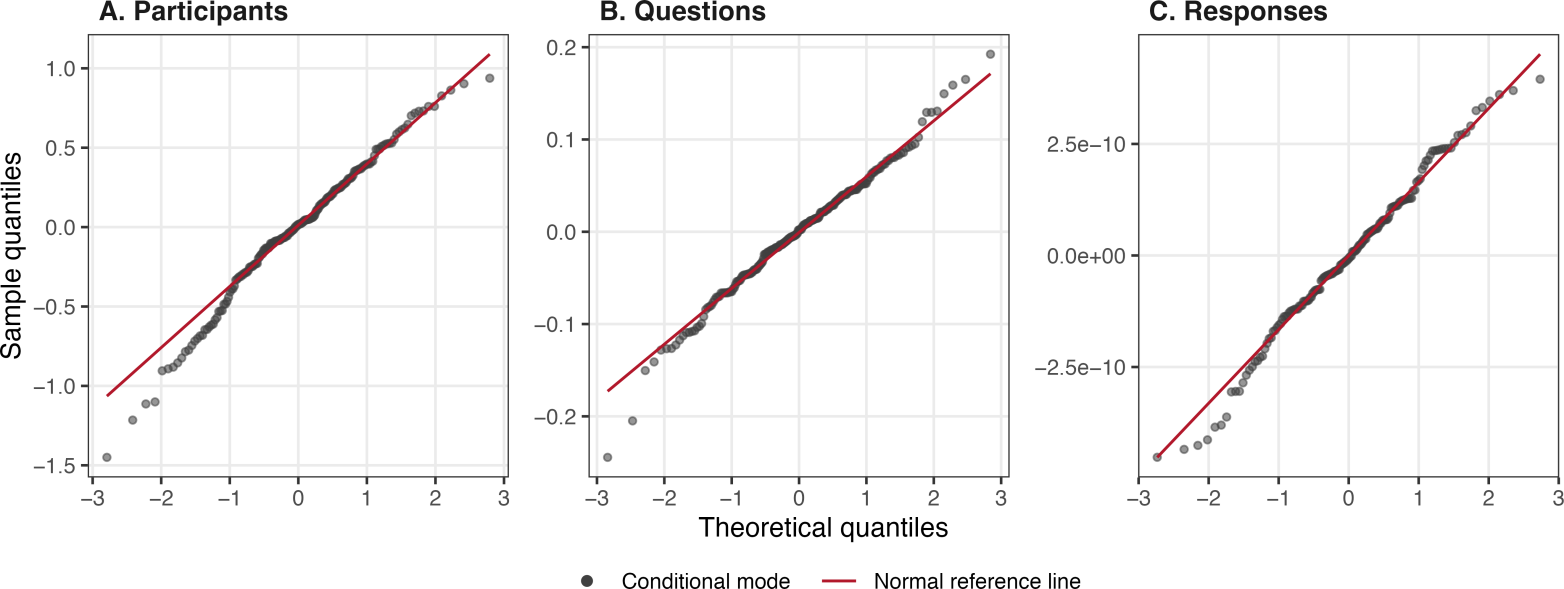
Quantile-quantile plots of conditional modes of random intercepts for (A) participants (N=191), (B) questions (N=40), and (C) responses (N=160).

We evaluated model diagnostics to verify that the structural assumptions of the CLMM were met. As illustrated by the quantile-quantile plots in Figure 1, the conditional modes of the random intercepts for participants (Figure 1A), questions (Figure 1B), and responses (Figure 1C) closely approximated a normal distribution, supporting the use of normally distributed random effects. We tested the proportional odds assumption using nominal likelihood-ratio tests within the fixed-effects specification. All predictors except Thoroughness (*P*<.001) satisfied this assumption. However, ordinal regression models are generally robust to isolated violations of proportional odds when the remaining predictors conform [54,55]. We therefore retained the standard model specification.

#### Power

We conducted an a priori power analysis using a Monte Carlo simulation to estimate the required sample size for the planned CLMM. We wrote a custom simulation script that generated 1000 synthetic datasets mirroring the study’s design: each dataset included 8 correlated predictors drawn from ordinal 1 to 5 distributions, with each predictor assigned a true odds ratio of 1.25. Within-participant dependence was modeled using a random intercept with an intraclass correlation coefficient of 0.25. Across simulations with a 2-sided α value of .05, results indicated that a minimum of 150 participants would be required to achieve 80% statistical power. To ensure sufficient power for the final model, we recruited a sample of 191 caregivers, yielding greater than 99% power to detect at least 1 significant predictor in the same simulation framework.

#### Qualitative Analysis

Each participant provided open-ended comments for every response they evaluated, describing what they liked, what they disliked, and why they preferred one response over the other in each vignette. This yielded 1146 comments across all vignette evaluations. Of these, 834 contained substantive feedback and were included in the analysis. Comments that were blank, off-topic, or too vague to code were excluded. Open-ended comments submitted in Spanish were translated into English using Google Translate and verified by a bilingual researcher.

We analyzed these comments using inductive thematic analysis. Two researchers (RH and SC) independently coded an initial sample of 250 comments and iteratively developed a codebook through discussions until consensus was reached. We assessed interrater reliability on a separate sample of 100 comments coded independently by both researchers using the finalized codebook, yielding a Cohen κ of 0.68, indicating substantial agreement. The first author (RH) then coded the remaining comments using the finalized codebook.

#### Data Exclusion

To ensure data quality, we designed 5 attention check questions and 2 honeypot questions (invisible to human participants but detectable by automated bots). Participants who failed more than 2 attention checks or triggered any honeypot were excluded from analysis. We set this threshold to filter out inattentive or automated responses while accommodating reasonable human error.

## Results

### User Statistics

After quality checks and attention screening, our final sample included 191 participants, who together evaluated 573 vignettes and provided 1146 evaluations. The participants represented a diverse demographic, as shown in Table 2.

**Table 2.** Demographic and background information.

Variables	Count, n (%)
Sex	
Female	115 (60.2)
Male	76 (39.8)
Race/ethnicity	
White	132 (69.1)
Black	35 (18.3)
Asian	4 (2.1)
Mixed	12 (6.3)
Other	8 (4.2)
Employment	
Full-time	106 (55.5)
Part-time	30 (15.7)
Not in paid work (eg, retired)	14 (7.3)
Unemployed	6 (3.1)
Unknown	35 (18.3)
Education	
Doctorate	12 (6.3)
Master’s degree	58 (30.4)
Bachelor’s degree	68 (35.6)
Some college or associate degree	43 (22.5)
High school diploma or equivalent	10 (5.2)
Fluent languages (multiple choice)	
English	191 (100)
Spanish	52 (27.4)
Other	10 (5.2)
Survey language	
English	151 (79.1)
Spanish	40 (20.9)
Online medical resources	
Curated website (eg, WebMD)	129 (67.5)
AI^a^ chatbots	137 (71.7)
Web search	169 (88.5)
Social forum	72 (37.7)
Other	5 (2.6)
None	0 (0)
Age (years), mean (SD)	40.17 (10.18)

a AI: artificial intelligence.

### RQ1: How Do Caregivers Perceive the Importance of Each Evaluation Dimension?

**Figure 2.**
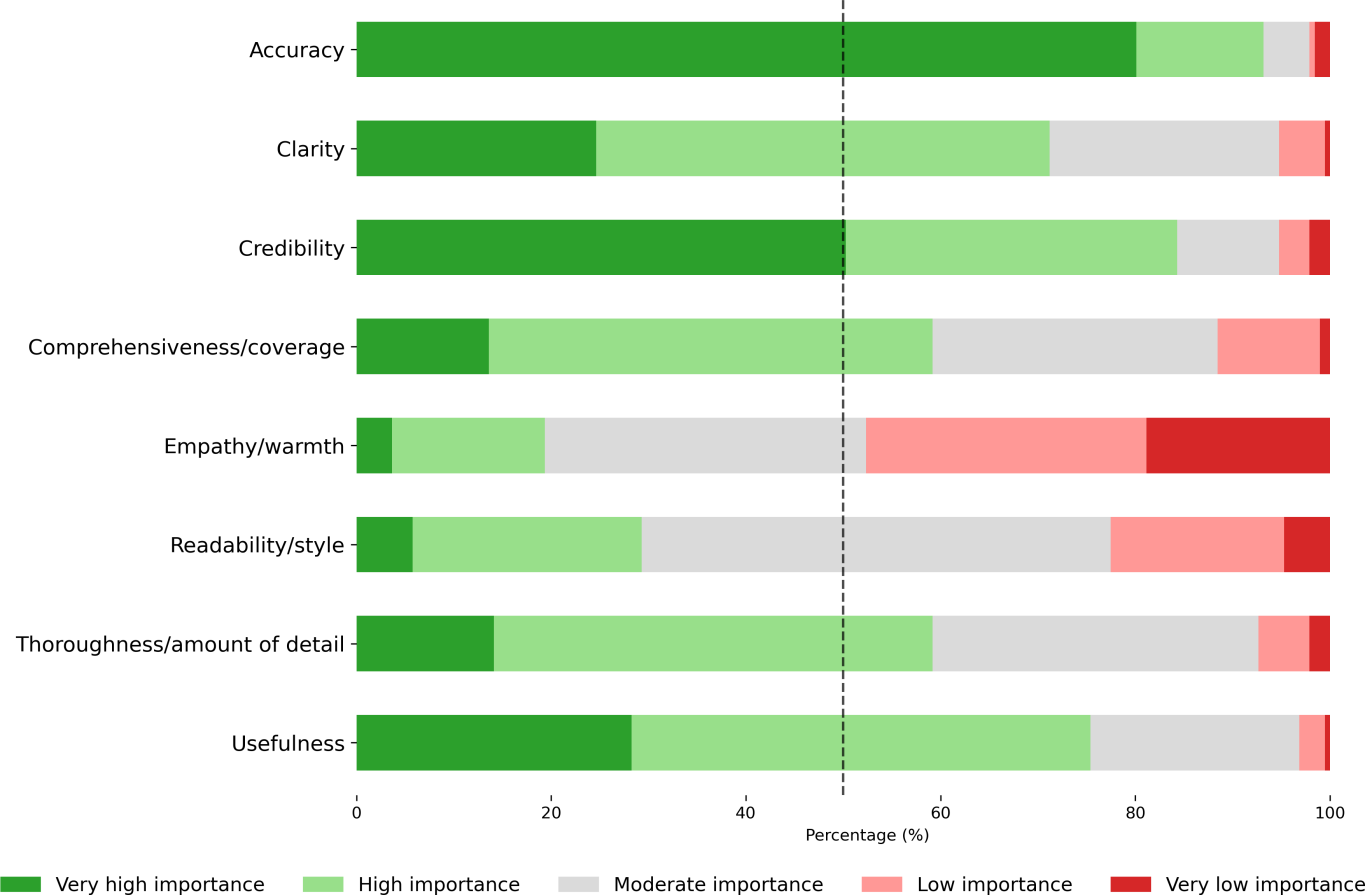
Caregivers’ ratings for the importance of 8 evaluation dimensions.

Figure 2 presents caregivers’ importance ratings across the 8 evaluation dimensions. Since these ratings were collected before participants were exposed to any vignettes with example chatbot responses, they capture respondents’ prior expectations of medical AI.

Accuracy and Credibility were rated as the most important dimensions, with over 80% of respondents assigning them a “High Importance” or “Very High Importance” rating. Usefulness, Thoroughness, Comprehensiveness, and Clarity formed a middle tier, with roughly 50%-70% of caregivers rating them in the top 2 importance levels. Empathy/Warmth received the lowest importance ratings, with nearly half of the respondents rating it as “Low Importance” or “Very Low Importance.” Readability/Style also received mixed feedback, with approximately 25% of respondents rating it in the bottom 2 importance levels.

All dimensions were rated at least “Moderately Important” by a majority of respondents (>50%), indicating that caregivers broadly recognize them as relevant criteria for assessing medical chatbot quality. However, the distribution is far from uniform. Caregivers placed the highest importance on factual qualities such as Accuracy and Credibility, followed by content-oriented qualities such as Usefulness, Clarity, Thoroughness, and Comprehensiveness*,* and the lowest importance on communicative qualities such as Empathy/Warmth and Readability/Style. As we show in the following sections, however, dimensions that most strongly predict caregivers’ overall satisfaction in practice do not align with this ordering.

### RQ2: How Satisfied Are Caregivers With Chatbot Responses Based on the Evaluation Framework?

**Figure 3.**
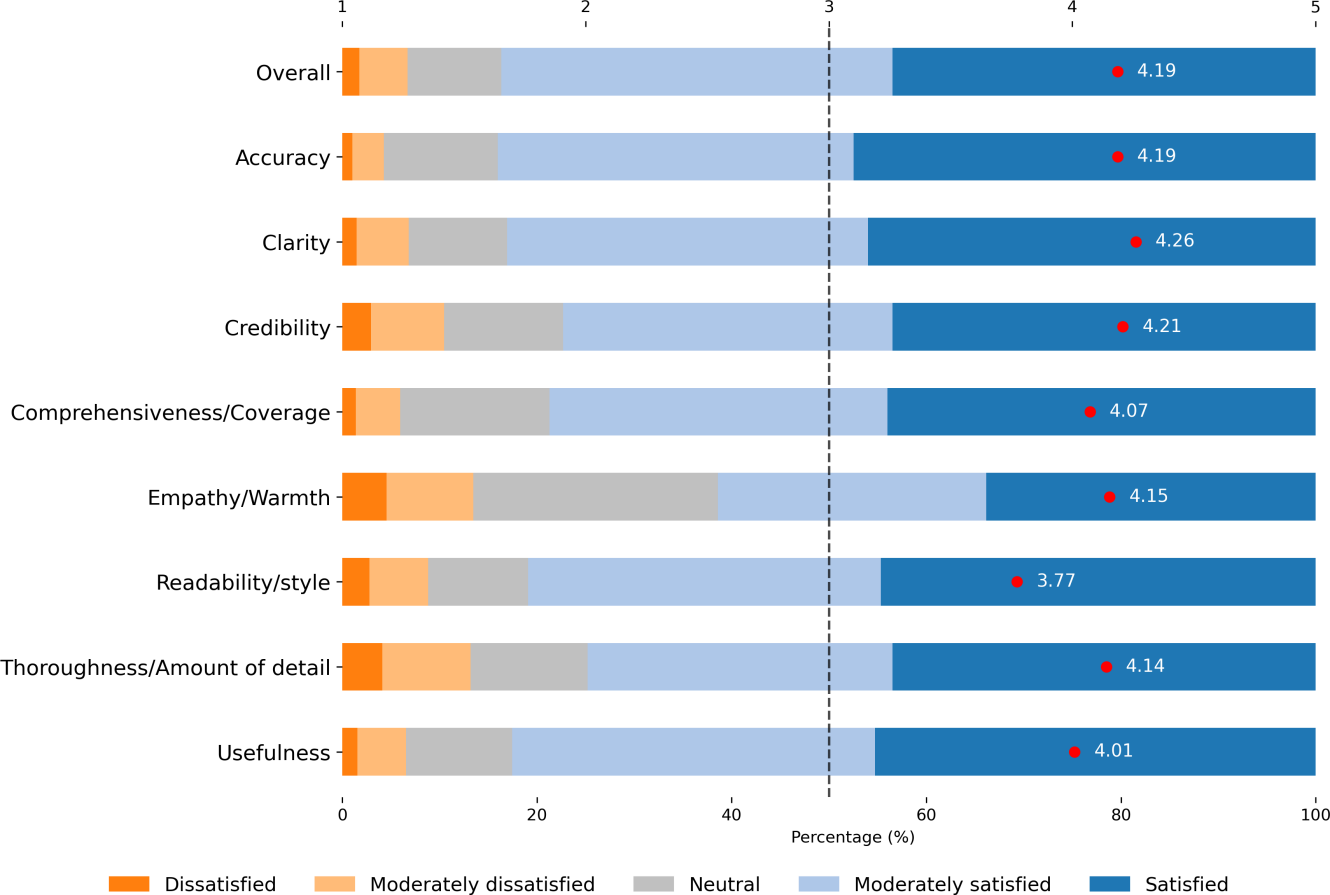
Caregivers’ satisfaction ratings across 8 evaluation dimensions.

Figure 3 shows caregivers’ satisfaction ratings across the 8 dimensions and overall. Ratings clustered toward the positive end of the scale: every dimension received a median of 4 out of 5, and mean scores ranged from 3.77 to 4.26. This concentration at the upper end of the scale suggests that GPT-4o’s responses were generally well received, although it also limits the range of variation available for distinguishing among dimensions.

Although ratings were generally positive, 3 dimensions stood out with more dispersed and more negative scores. Empathy/Warmth was a clear outlier, with the lowest mean score (3.77) and approximately 20% of ratings falling at “Moderately Dissatisfied” or below. Thoroughness (mean 4.01) and Credibility (mean 4.07) also showed wider spread than the remaining dimensions, which clustered between 4.14 and 4.26.

To examine whether these lower and more variable ratings reflected systematic disagreement among caregivers rather than uniform moderate dissatisfaction, we computed Van der Eijk’s agreement score for each dimension. This index quantifies the degree of consensus in a rating distribution on a scale from −1 to +1, with values near 0 indicating disagreement (ie, a uniform spread). We computed the score for each response and then averaged across responses. As shown in Table 3, Empathy/Warmth had the lowest mean agreement (μ=0.378), followed by Thoroughness (μ=0.474), while Accuracy (μ=0.636) and Usefulness (μ=0.548) showed the strongest consensus.

**Table 3.** Van der Eijk agreement scores for satisfaction ratings across dimensions.

Dimension	Agreement	
	Median	Mean
Accuracy	0.750	0.636
Usefulness	0.708	0.548
Comprehensiveness/Coverage	0.688	0.550
Clarity	0.682	0.528
Readability/Style	0.650	0.477
Credibility	0.643	0.503
Thoroughness/Amount of detail	0.625	0.474
Empathy/Warmth	0.500	0.378

Qualitative comments help explain this pattern. For Empathy/Warmth, participants did not give low ratings because the chatbot failed to produce empathetic responses. Rather, lower ratings mainly reflect participants’ different preferences toward AI communication styles. Some caregivers valued emotional acknowledgment, while others described AI-generated empathy as “artificial,” “unnecessary,” or “patronizing*”* [P50], and preferred a neutral or clinical tone. As a result, participants who preferred a neutral or professional tone tended to rate empathetic responses poorly.

### RQ3: How Does Each Dimension Influence the Overall Satisfaction?

Tables4  5 report the CLMM results, showing the odds ratio for each evaluation dimension’s association with overall satisfaction, controlling for the other dimensions.

*Usefulness* was the strongest predictor: each 1-point increase in Usefulness was associated with 2.53 times higher odds of a higher overall satisfaction (95% CI 1.95‐3.27; *P*<.001). Thoroughness was the second strongest (OR 2.15, 95% CI 1.69‐2.73; *P*<.001). Accuracy (OR 1.81, 95% CI 1.38‐2.36; *P*<.001) and Credibility (OR 1.52, 95% CI 1.19‐1.94; *P*=.002) were also significant predictors but notably weaker than Usefulness and Thoroughness, despite being rated as the 2 most important dimensions in RQ1. In contrast, Readability/Style (OR 1.75, 95% CI 1.43‐2.13; *P*<.001) and Empathy/Warmth (OR 1.48, 95% CI 1.27‐1.72; *P*<.001) were both significantly associated with satisfaction despite being rated among the least important dimensions in RQ1. This suggests that presentation-level qualities, that is, how information is worded and formatted, also meaningfully shape caregivers’ evaluations even when caregivers themselves do not see them as priorities.

**Table 4.** CLMM^a^ results: association of each evaluation dimension with overall satisfaction^b^.

	Odds ratio (95% CI)		SE	*z* value	*P* value
Accuracy	1.81 (1.38‐2.36)		0.13629	4.335	<.001
Clarity	1.45 (1.14‐1.86)		0.12596	2.968	.006
Comprehensiveness	1.05 (0.82‐1.33)		0.12236	0.377	.71
Credibility	1.52 (1.19‐1.94)		0.12485	3.353	.002
Empathy/Warmth	1.48 (1.27‐1.72)		0.07679	5.064	<.001
Readability/Style	1.75 (1.43‐2.13)		0.10120	5.522	<.001
Thoroughness	2.15 (1.69‐2.73)		0.12248	6.251	<.001
Usefulness	2.53 (1.95‐3.27)		0.13238	7.000	<.001

a CLMM: Cumulative Link Mixed Model.

b Odds ratios, 95% CIs, and Holm-corrected *P* values are reported.

**Table 5.** Random-effects variances and standard deviations.

Random effects	Variance	SD
Participant	0.53300	0.7301
Question	0.07423	0.2725
Response	1.66e-10	1.29e-05

Comprehensiveness was the only dimension whose effect was not statistically significant (OR 1.05, 95% CI 0.82‐1.33; *P*=.71). One possible explanation is that its effect is partially absorbed by Thoroughness, which is conceptually adjacent (both concern the amount of information provided, although Thoroughness is about the depth of reasoning while *Comprehensiveness* is about the breadth and coverage). Alternatively, as caregivers’ open-ended comments suggest (detailed in RQ4), participants may have associated comprehensive responses with excessive length, viewing them as a liability rather than an asset. Participants frequently described preferred responses as “straight to the point” and criticized longer responses as unnecessarily exhaustive, hindering their ability to extract useful information quickly.

Among the random effects, participant-level variance (0.533) was substantial, confirming that caregivers differed meaningfully in their baseline rating tendencies. Question-level variance was smaller (0.074), and response-level variance was near zero (1.66×10⁻¹⁰), suggesting that once the 8 dimension ratings are accounted for, little variation remained across specific response texts.

To check whether results were sensitive to survey language, we fitted the CLMM using only those participants who took the English survey (n=151). The direction and significance of all predictors were identical to those from the full sample, suggesting that the findings were consistent across language groups.

### RQ4: What Evaluation Considerations Emerge Beyond Existing Frameworks?

To uncover potential nuances that existing frameworks might have overlooked, we conducted an inductive thematic analysis of 1146 open-ended comments in which participants described what they liked and disliked about each chatbot response and explained their preference. This analysis produced 9 categories of qualities that caregivers desire (Table 6; 443 positive comments) and 9 categories of concerns (Table 7; 415 negative comments). Several categories corresponded to the predefined evaluation dimensions (eg, Credible and Lack of Empathy/Warmth), confirming that the framework captures qualities that caregivers value. However, others surfaced considerations not represented in the existing dimensions, particularly around the type of detail caregivers want, preferences for conversational interaction, and context-dependent reactions to tone and disclaimers. We organize the findings in the subsections that follow around these 4 emergent themes.

**Table 6.** Desired qualities identified from thematic analysis of positive open-ended comments (N=443).

Desire	Description	Count
Straight to the point	The response cuts straight to answering the question directly, without unnecessary information.	159
Appropriate tone	The response shows warmth and takes on a natural, comforting, and human-like tone.	118
Readable	The response is well organized, making it easy to read and follow.	97
Detailed/Comprehensive	The response provides detailed information, making it feel complete.	84
Practical/Actionable	The response is useful, actionable, and suggests immediate actions to follow.	81
Clear/Accessible	The response uses easy-to-follow language, making it clear and accessible to nonprofessional audiences.	56
Credible	The response uses various sources to support its claim, making it appear credible and trustworthy.	47
Concise/Right amount of information	The response is concise yet complete, containing just the right amount of information.	47
Disclaimer	The response provides accurate warnings or cautions to urge users to engage their own judgment.	23

**Table 7.** Concerns identified from thematic analysis of negative open-ended comments (N=415).

Concerns	Description	Count
Too long/Not straight to the point	The response is too long, making it difficult to locate useful information.	120
Lack of detail	The response lacks specific details.	116
Lack of empathy/Warmth	The response feels cold and lacks warmth.	77
Lack of actionability	The response does not provide practical and useful advice about the next step or indicate the urgency.	77
Lack of readability	The response is poorly organized, contains jargon, and is overall hard to follow and understand.	73
Lack of credibility	The response is not supported by credible sources and appears suspicious.	49
Lack of explanation	The response lacks explanations for the information, assumptions, or suggestions.	46
Overly empathetic	The response comes off too conversational and personal, making it seem insincere and fake.	37
Untimely disclaimer	Response makes a broad use of warnings or gives unwanted disclaimers.	18

### The Right Kind of Detail: Why Caregivers Want Less but Need More?

Caregivers frequently praised responses for being *“*straight to the point*”* (159/443, 35.9%), noting that concise answers helped them identify and “absorb” what mattered more quickly [P127], especially when they were “busy or stressed” [P72] or making decisions under time pressure [P56]. Correspondingly, excessive length was the most frequent complaint (120/415, 28.9%). Many caregivers reported feeling overwhelmed or confused when presented with too much information at once. P28, for example, explained that trying to process a lengthy response while tending a sick child left them “overwhelmed by too much fluff” and thus unable to focus on urgent decisions. P72 put it plainly:

*I would rather have concise medical information to help address my needs quickly without having to go through medical school, learn jargon to understand*.

[P72]

Yet caregivers simultaneously raised concerns about insufficient detail (116/415, 28%). However, closer examination of these comments suggested that “lack of detail” was often used as a catch-all complaint rather than a literal request for longer or more exhaustive answers. When caregivers flagged a response as lacking detail, it was often because a specific type of information felt missing, not that they actually needed deeper or broader explanations. We identified 3 recurring information needs masked behind the complaint of “lack of detail” (Textbox 1):

Textbox 1. **Recurring information needs.**

1. Actionable guidance: Caregivers wanted chatbots to move beyond explanation and provide concrete next steps, particularly for triaging “severity” [P114] and determining “when to seek medical help” [P88]. Several participants felt uneasy that responses left out a “specific time frame” [P12] and “symptoms or signs to watch for” [P88], which they considered essential for making confident decisions [P125]. They also wanted concrete advice about what to do immediately and what could be safely “troubleshoot at home” [P54], which, as one participant noted, “could have reassured concerned parents” [P136]. Even when remedies were listed, caregivers noted the absence of “direct guidance” [P122] on how to carry them out in practice. At its most serious, missing urgency cues carries real stakes: P150 remarked that vague guidance could lead caregivers to delay care when, under normal judgment, they “should have taken my child to the emergency room already.”
2. Credible references: Missing sourcing was a major concern in negative feedback (49/415, 11.8%), while citing authoritative references was correspondingly praised in positive feedback (47/443, 10.6%). Caregivers wanted to know where the information came from, and when a response provided no indication of its source, they were less willing to trust or act on it [P22]. Even when citing primary literature was not feasible, caregivers felt that pointing to a recognized authority would suffice. P37 explained: “I prefer to know where this information is coming from. But, if I can’t, it would be good if they could refer me to a credible source, like WebMD, CDC, or NIH, for more information on this topic” [P37].
3. Logical reasoning: Bare recommendation lists without any rationale were flagged as difficult to trust or act on (46/415, 11.1%). Caregivers did not simply want to be told what to do; they wanted to understand why, so they could exercise their own judgment. This aligns with broader findings on explainable AI in clinical decision support, where transparency in reasoning has been shown to enhance user trust [56]. P3 complained that a response “did list exactly what I requested, but it didn’t elaborate,” and P42 put it more bluntly: “It was too short and not credible, seemed like the Temu answer to an Amazon question.” Without explanation, even technically correct advice felt hollow and unconvincing.

Some participants pointed toward a resolution: rather than receiving all information at once, they preferred a concise initial answer with the option to go deeper through follow-up questions. P14 explained, “I get my concise answer, and if I felt the need to go deeper, I would proceed as so myself.” Others described frustration at being “thrown int*o*” [P131] answers that tried to “pack everything” [P109] into a single response. P28 made the point most directly: “It’s not like the user only gets one chance to ask a question, they can ask follow-up questions!”

### Context-Aware Tone and User Preference

Tone was a prominent theme in open-ended comments. In positive comments, out of 443, 118 (26.6%) praised responses for warmth and a natural, human-like tone. In negative comments, out of 415, 77 (18.6%) criticized responses as too cold, while out of 415, 37 (8.9%) described them as overly empathetic. These opposing complaints reflect the same polarization observed in RQ2, in which Empathy/Warmth had the lowest agreement score among respondents. The qualitative data revealed 3 distinct orientations toward empathetic tone.

#### Warmth as Reassurance

Some caregivers responded positively to empathetic language, particularly when the topic felt personal or alarming. P145 appreciated a tone that felt “warm and empathetic,” noting that it made “the information feel more personal and supportive.” P92 similarly valued responses that felt “caring and relatable,” and P70 noted that empathetic language made them feel that their “emotions and feelings were considered.” For caregivers under stress, this extended to practical reassurance: P52 appreciated responses that encouraged parents to “trust their instincts,” describing this as comforting in high-pressure situations. For these participants, empathy was not merely stylistic; it signaled that the chatbot understood the emotional stakes.

#### Restraint as Professionalism, Depending on Context

Other caregivers preferred an empathetic tone to be modulated by the situation. When the question felt urgent or factual, warmth could dilute clarity or authority, making the response appear less professional and less trustworthy. P50 explained that in high-urgency situations, they “want to trust and believe what I’m being told, not have the AI hemming and hawing and sounding more like a friend at a kitchen table.” P56 similarly preferred a “crisp, professional tone” that avoided “unnecessary familiarity” [P37]. At the same time, P146 noted that a casual voice “might come off as slightly too emotionally focused for someone seeking clear, medically grounded information.” These participants did not reject empathy categorically but considered it inappropriate when the priority was clear, medically grounded information.

#### Rejection of AI Empathy

A total of 18 participants explicitly rejected any displays of empathy from AI. Their concerns clustered around 3 issues. First, credibility: P37 found familiar or personal language so out of place that it led them to doubt the information itself, noting “I don’t see a medical professional speaking this way.” Second, efficiency: P12 remarked that “too much extra may take more time than necessary, hindering my ability to get quick help for my child,” and P28 put it more bluntly: “we need to be talked to like we are clueless, but not pandered to with a bunch of lame touchy-feely empathetic stuff.” Third, sincerity: several participants described AI-generated sympathy as “super fake” [P83] or “patronizing” [P149], with P50 adding that it “makes me distrust the information because it feels like less of an authority I should take seriously.”

### Timely Disclaimers and Urgency Cues

Caregivers paid close attention to disclaimers and urgency cues, but their reactions were not uniformly positive or negative. The same language that reassured one caregiver undermined another’s confidence, depending largely on the context in which it appeared.

When responses involved treatment suggestions, dosage guidance, or potentially serious symptoms, caregivers often welcomed clear disclaimers. In these contexts, disclaimers such as “I am not a doctor; please consult a medical professional” were often interpreted as responsible and trust-building. P131 noted that they liked it when a response “suggested this is something that needs to be discussed with a human,” as it prompted them to exercise their own judgment rather than act solely on the chatbot’s advice. For higher-stakes content, a well-placed disclaimer signaled that the chatbot recognized the limits of its role, which participants perceived as “responsible” [P156].

In lower-stakes situations, however, the same language could have the opposite effect. When a response relayed basic facts, routine disclaimers were often seen as redundant, already “assumed” [P37], or even “patronizing” [P149]. Some participants even felt dismissed, as if the chatbot “didn’t have time to take my question seriously” [P61] or had little substantive information to offer: “this small amount of info would infer that...there’s no way to know for sure until you see your healthcare provider” [P37]. P27 similarly described blanket usage of disclaimers as “repetitive,” while P152 noted that they “reduce confidence in the advice” by making every response feel equally uncertain, regardless of actual risk.

Caregivers also wanted responses to communicate urgency more clearly. Several participants felt that the chatbot did not provide enough guidance in distinguishing situations that warranted immediate attention from those appropriate for watchful waiting. P136 noted that a response “should mention when to seek medical help if the issue persists, which could have reassured concerned parents,” and P119 similarly pointed out that caregivers “might be unsure about the severity of certain symptoms” without explicit guidance. At the same time, caregivers were equally wary of responses that leaned toward alarm. They preferred guidance that neither catastrophized, “unlike every other health thing you look up in a Google Search” [P50], nor minimized symptoms but instead helped them calibrate their own response. P28 captured this balance well:

*I love them dearly and don' t want anything bad to happen to them, but also don' t want to be crazy and overbearing and rush them to the hospital every time they sneeze*.

[P28]

## Discussion

### Principal Findings

Our findings highlight 3 gaps in how medical chatbots are currently evaluated and designed. First, what caregivers mean by “detail” does not map neatly onto dimensions such as Thoroughness or Comprehensiveness. Existing evaluation frameworks lack a way to capture how efficiently a response delivers what users need in decision-making. Second, existing frameworks evaluate individual responses in isolation but do not assess how well a chatbot supports an ongoing conversation. Third, qualities such as empathetic tone and medical disclaimers are not universally beneficial but depend on both individual preferences and situational context, which current evaluation protocols do not account for.

### More Information Is Not Better Information

RQ4 shows that caregivers simultaneously complained about excessive length and insufficient detail, but as our qualitative analysis indicated, these complaints targeted different qualities. When caregivers asked for more “detail,” they were not simply asking for longer, deeper, or more exhaustive explanations. They were asking for responses that better equipped them to make confident decisions. Specifically, they wanted to know what symptoms to watch for, why a recommendation made sense, and where the information came from. Conversely, when they complained about length, they were criticizing the low density of useful information for immediate decision-making.

Existing evaluation dimensions are not designed to capture this distinction. Thoroughness measures the depth of explanation, while Comprehensiveness measures the breadth of topics covered. Neither assesses whether a response delivers the specific information a caregiver needs to take action. The CLMM results support this interpretation: Comprehensiveness had no significant effect on satisfaction (OR 1.05, 95% CI 0.82-1.33), while Usefulness (the dimension closest to actionable guidance in our framework) was the strongest predictor (OR 2.53, 95% CI 1.95-3.27). After accounting for the other dimensions, covering more ground did not, on its own, increase satisfaction; what mattered most was whether responses helped caregivers act confidently. We therefore propose that future evaluation frameworks include such a dimension that captures how efficiently a response delivers the specific information caregivers desire in decision-making, for example, warning signs, concrete next steps, references from authoritative sources, and so on.

A related concern is that some sources caregivers perceived as credible in their comments (eg, WebMD) are not always accurate or the most trustworthy. Yet, current general-purpose chatbots do not systematically vet the sources they draw from, which may include outdated or commercially biased content. The responsibility for source quality cannot rest with the caregiver. Instead, chatbots should actively review and vet sources, directing users to authoritative, peer-reviewed, or government-vetted sources. Emerging platforms such as OpenEvidence [57], which ground clinical responses in peer-reviewed literature, offer a model for adapting such evidence-anchoring strategies for caregiver-facing chatbots.

### From Monologue to Dialogue

As reported in RQ4, some caregivers described a natural resolution to the tension between conciseness and detail: start with a short answer and let them ask for more. Rather than passively receiving a dense block of information at once, they preferred to seek exactly what they needed through follow-up questions. This highlights that caregivers do not evaluate a single chatbot response in isolation. A response that delivers all possible information upfront may technically be thorough, but it can frustrate users who want to control the pace and depth of the information they receive.

None of the evaluation frameworks we reviewed assesses this conversational dynamic. Dimensions such as Thoroughness and Comprehensiveness score the content of a single response. They do not measure whether a chatbot helps users navigate a multiturn exchange, for example, by offering to elaborate on severity thresholds or underlying reasoning. As a result, future evaluation protocols should consider how well a chatbot supports multiturn conversations [58,59].

From a design perspective, this suggests that chatbots should treat their initial response as an entry point rather than a comprehensive, stand-alone answer. As multiple caregivers in our sample described, they preferred responses that invited follow-up, for instance, by signaling what additional information was available, such as elaboration on urgency thresholds, reasoning behind a recommendation, or pointers to additional resources. This approach fosters an ongoing exchange where caregivers can efficiently gather what they need without being overwhelmed by information they did not ask for.

### One Size Does Not Fit All: Tone, Trust, and Situational Sensitivity

RQ3 shows that Empathy/Warmth significantly influenced caregivers’ satisfaction with medical chatbots. At the same time, RQ2 showed that caregivers varied widely in their perceptions of an appropriate tone. The qualitative findings in RQ4 suggest that these differences reflected both personal preferences and situational context, and they challenge a common assumption in chatbot design: that warmer, more human-like communication is universally beneficial [48]. A substantial body of work has focused on building empathetic chatbot personas on the premise that emotional expressiveness builds trust and facilitates human-AI interaction [60-62]. Our data suggest that this holds for some caregivers in some situations but actively backfires for others. Among the 18 caregivers in our sample who rejected empathetic language from AI entirely, warm tone did not just fail to help; it undermined the chatbot’s perceived credibility. These caregivers viewed emotional expressiveness from an AI system as performative or manipulative, suggesting that for a meaningful subset of users, optimizing for warmth across the board risks eroding rather than building trust.

Disclaimers and urgency cues followed a similar context-dependent pattern. Disclaimers were helpful when they signaled the limits of the chatbot’s competence but frustrating when they felt routine or unnecessary, especially in lower-stakes scenarios. Caregivers also valued urgency cues that helped them monitor, triage, and manage symptoms at home but were wary of responses that leaned toward alarm. In both cases, the issue was not the presence of these features but their blanket use across different situational contexts.

The practical implication is the same across all 3 qualities: evaluation frameworks should move beyond treating Empathy/Warmth, disclaimers, and urgency cues as monotonic qualities in which higher is always better. The relevant question is not “How empathetic is this response?” or “Does this response include a disclaimer?” but whether these features are appropriate, given the user and the situation. An empathetic response may be excellent for one caregiver but alienating for another. A disclaimer may build trust for a dosage question and erode it for a question about cold symptoms. Future systems could support this by allowing users to indicate their communication preferences and by adjusting tone and disclaimer use based on the clinical context of the question.

### Comparison With Prior Work

Our findings both validate and extend prior work on the evaluation of medical chatbots. The face validity we observed for established dimensions aligns with the theoretical frameworks proposed by Tam et al [29] and Abbasian et al [32], confirming that these researcher-derived dimensions do resonate with end users. However, our CLMM results show that these dimensions differ substantially in their predictive strength. Usefulness was the strongest predictor of satisfaction (OR 2.53, 95% CI 1.95-3.27), while Comprehensiveness had no significant effect (OR 1.05, 95% CI 0.82-1.33). These findings challenge the implicit assumption in most frameworks that all dimensions contribute comparably to perceived quality and provide empirical evidence on their relative importance. This is an aspect that prior frameworks, largely derived from literature reviews and expert input, have not addressed.

The tension we identified between brevity and detail echoes findings from broader health information-seeking literature [11,63]. Still, our study provides more granular evidence about which specific types of detail caregivers need (actionability, credibility markers, and explanations) versus what they find burdensome (background information and exhaustive coverage). This specificity can guide more targeted response optimization.

Our findings on empathy diverge from a common design assumption in chatbot research: that warmer, more human-like communication is uniformly beneficial. An extensive body of work has focused on building empathetic chatbot personas on the premise that emotional expressiveness builds trust and improves the user experience [49,64,65]. Our data suggest that this assumption does not always hold, as some caregivers reject AI-generated empathy. This aligns with Seitz [66], who found that empathetic expressions in health care chatbots can reduce perceived authenticity, suppressing the trust gains that warmth would otherwise produce. Our qualitative findings converge on a similar point: caregivers who rejected AI empathy tended to perceive it as performative and preferred a professional, information-focused tone instead. The challenge for designers, therefore, is not whether to include empathy but when and how much, and for whom.

The importance of risk-calibrated disclaimers has received limited attention in the medical chatbot literature. Most systems currently apply disclaimers uniformly, likely driven by legal concerns. Our evidence suggests that this practice may be counterproductive, reducing trust in low-risk situations where disclaimers feel patronizing while potentially being insufficient in high-risk situations where more specific warnings are needed. This points toward a need for disclaimer strategies that scale with actual medical risk rather than being applied as legal boilerplate.

### Limitations

Several limitations should be considered when interpreting our findings. First, our study used GPT-4o as the sole source of chatbot responses. While GPT is one of the most widely used AI chatbots, caregivers in practice may encounter AI-generated content through other channels, such as Google search summaries. Evaluations of responses from different models or platforms may yield different patterns. Relatedly, our vignette design presented responses as static text rather than as part of a live conversation. This allowed for controlled comparisons across communication styles but did not capture how caregivers would interact with a chatbot in real time, where follow-up questions and iterative exchanges could shape their experience differently.

Second, our sample, while diverse in age, education, and employment, was predominantly White (132/191, 69.1%) and limited to US-based caregivers. Perceptions of empathy, authority, and an appropriate medical communication style vary across cultural and ethnic groups, and our findings may not generalize to other populations. Similarly, Prolific participants tend to be digitally literate and experienced with online surveys. Caregivers with lower digital health literacy may place greater emphasis on clarity and readability, respond differently to empathetic tone or disclaimers, and be less equipped to evaluate AI-generated content critically. Future work should recruit from clinical settings or community health programs and from more culturally diverse populations to examine how these factors moderate the patterns we observed.

Third, our study focused exclusively on pediatric infectious diseases, which are common, acute conditions. Caregivers managing chronic illnesses or navigating more complex medical decisions may weigh evaluation dimensions differently. We also did not control for contextual factors within the questions themselves. Emergency queries likely elicit different evaluation criteria than routine information requests, yet all questions were analyzed uniformly. Future work should systematically examine how clinical urgency and decision stakes influence the importance of the dimensions.

Fourth, the mixed reactions to empathetic responses raise interpretive questions. We cannot determine from our data alone whether negative reactions to AI empathy stem from perceived insincerity of the specific responses or from a more fundamental resistance to AI expressing emotions at all. A Wizard-of-Oz study, in which participants are unaware whether they are interacting with a human or an AI, would help disentangle these factors.

Fifth, participant eligibility was self-reported through Prolific without independent verification of parental status. However, because pediatric infectious diseases are common conditions familiar to most adults with caregiving experience, this limitation is unlikely to affect our findings meaningfully.

Finally, the pronounced right skew in satisfaction ratings may reflect measurement constraints. Our 5-point scale may have lacked sufficient granularity to capture nuanced differences at the higher end of the distribution. Future studies at larger scales could reveal greater variance in evaluations and enable finer-grained analysis of what distinguishes satisfactory from highly satisfactory responses.

### Conclusions

This study provides empirical evidence that established evaluation dimensions differ substantially in their predictive power for caregiver satisfaction with medical AI chatbots. Usefulness was the strongest driver of satisfaction, while Comprehensiveness did not significantly predict satisfaction. Qualitative findings revealed that caregivers want actionable guidance, credible references, and clear reasoning rather than exhaustive detail, and prefer concise initial answers with the option to ask follow-up questions. Our results also challenge the assumption that empathetic tone and medical disclaimers are broadly preferable, as both qualities proved context-dependent, helping in some situations while undermining trust in others. These findings suggest that future evaluation frameworks should empirically weight dimensions rather than treating them as interchangeable, assess how well chatbots support multiturn conversations, and account for individual and situational variation in communication preferences.

## Abbreviations

AI: artificial intelligence
CLMM: Cumulative Link Mixed Model
RQ: research question
VIF: variance inflation factor

## References

[1] Meskó B, Topol EJ (2023). The imperative for regulatory oversight of large language models (or generative AI) in healthcare. NPJ Digit Med.

[2] Thirunavukarasu AJ, Ting DSJ, Elangovan K, Gutierrez L, Tan TF, Ting DSW (2023). Large language models in medicine. Nat Med.

[3] Kühne S, Jacobsen J, Legewie N, Dollmann J (2025). Attitudes toward AI usage in patient health care: evidence from a population survey vignette experiment. J Med Internet Res.

[4] Tudor Car L, Dhinagaran DA, Kyaw BM (2020). Conversational agents in health care: scoping review and conceptual analysis. J Med Internet Res.

[5] Abbas SR, Seol H, Abbas Z, Lee SW (2025). Exploring the role of artificial intelligence in smart healthcare: a capability and function-oriented review. Health Care (Don Mills).

[6] Ayers JW, Poliak A, Dredze M (2023). Comparing physician and artificial intelligence chatbot responses to patient questions posted to a public social media forum. JAMA Intern Med.

[7] Liu J, Wang C, Liu S (2023). Utility of ChatGPT in clinical practice. J Med Internet Res.

[8] Shahsavar Y, Choudhury A (2023). User intentions to use ChatGPT for self-diagnosis and health-related purposes: cross-sectional survey study. JMIR Hum Factors.

[9] Alain G, Crick J, Snead E, Quatman-Yates CC, Quatman CE (2025). Evaluating user interactions and adoption patterns of generative AI in health care occupations using Claude: cross-sectional study. J Med Internet Res.

[10] Semigran HL, Linder JA, Gidengil C, Mehrotra A (2015). Evaluation of symptom checkers for self diagnosis and triage: audit study. BMJ.

[11] Powell J, Inglis N, Ronnie J, Large S (2011). The characteristics and motivations of online health information seekers: cross-sectional survey and qualitative interview study. J Med Internet Res.

[12] Ghorashi N, Ismail A, Ghosh P, Sidawy A, Javan R (2023). AI-powered chatbots in medical education: potential applications and implications. Cureus.

[13] Kaarre J, Feldt R, Keeling LE (2023). Exploring the potential of ChatGPT as a supplementary tool for providing orthopaedic information. Knee Surg Sports Traumatol Arthrosc.

[14] Kuroiwa T, Sarcon A, Ibara T (2023). The potential of ChatGPT as a self-diagnostic tool in common orthopedic diseases: exploratory study. J Med Internet Res.

[15] Ayo-Ajibola O, Davis RJ, Lin ME, Riddell J, Kravitz RL (2024). Characterizing the adoption and experiences of users of artificial intelligence-generated health information in the United States: cross-sectional questionnaire study. J Med Internet Res.

[16] Meskó B (2023). The impact of multimodal large language models on health care’s future. J Med Internet Res.

[17] Goodman RS, Patrinely JR, Stone CJ (2023). Accuracy and reliability of chatbot responses to physician questions: Abstract. JAMA Network Open.

[18] Sivarajkumar S, Kelley M, Samolyk-Mazzanti A, Visweswaran S, Wang Y (2024). An empirical evaluation of prompting strategies for large language models in zero-shot clinical natural language processing: algorithm development and validation study. JMIR Med Inform.

[19] Gilson A, Safranek CW, Huang T (2023). How does ChatGPT perform on the United States Medical Licensing Examination (USMLE)? the implications of large language models for medical education and knowledge assessment. JMIR Med Educ.

[20] Walker HL, Ghani S, Kuemmerli C (2023). Reliability of medical information provided by ChatGPT: assessment against clinical guidelines and patient information quality instrument. J Med Internet Res.

[21] Xue Z, Zhang Y, Gan W, Wang H, She G, Zheng X (2024). Quality and dependability of ChatGPT and DingXiangYuan forums for remote orthopedic consultations: comparative analysis. J Med Internet Res.

[22] Choudhury A, Shamszare H (2023). Investigating the impact of user trust on the adoption and use of ChatGPT: survey analysis. J Med Internet Res.

[23] Wei Q, Yao Z, Cui Y, Wei B, Jin Z, Xu X (2024). Evaluation of ChatGPT-generated medical responses: a systematic review and meta-analysis. J Biomed Inform.

[24] Li J (2023). Security implications of AI chatbots in health care. J Med Internet Res.

[25] Chang Y, Wang X, Wang J (2024). A survey on evaluation of large language models. ACM Trans Intell Syst Technol.

[26] Nori H, King N, McKinney SM, Carignan D, Horvitz E (2023). Capabilities of GPT-4 on medical challenge problems. arXiv.

[27] Bedi S, Liu Y, Orr-Ewing L (2025). Testing and evaluation of health care applications of large language models: a systematic review. JAMA.

[28] Degachi C, Dhar U, Niforatos E, Kortuem G (2025). Towards a domain expert evaluation framework for conversational search in healthcare. CHI EA ’25: Proceedings of the Extended Abstracts of the CHI Conference on Human Factors in Computing Systems.

[29] Tam TYC, Sivarajkumar S, Kapoor S (2024). A framework for human evaluation of large language models in healthcare derived from literature review. NPJ Digit Med.

[30] van der Lee C, Gatt A, van Miltenburg E, Krahmer E (2021). Human evaluation of automatically generated text: current trends and best practice guidelines. Comput Speech Lang.

[31] Singhal K, Azizi S, Tu T (2023). Large language models encode clinical knowledge. Nature.

[32] Abbasian M, Khatibi E, Azimi I (2024). Foundation metrics for evaluating effectiveness of healthcare conversations powered by generative AI. NPJ Digit Med.

[33] Sallam M, Al-Salahat K, Al-Ajlouni E (2023). ChatGPT performance in diagnostic clinical microbiology laboratory-oriented case scenarios. Cureus.

[34] Kang C, Lee SW, Jung H, Bae Y (2025). Psychiatric morbidity following intestinal infectious diseases: a nationwide cohort study in South Korea. Stress Health.

[35] Bianco A, Zucco R, Nobile CGA, Pileggi C, Pavia M (2013). Parents seeking health-related information on the internet: cross-sectional study. J Med Internet Res.

[36] Peer E, Brandimarte L, Samat S, Acquisti A (2017). Beyond the Turk: alternative platforms for crowdsourcing behavioral research. J Exp Soc Psychol.

[37] Palan S, Schitter C (2018). Prolific.ac—a subject pool for online experiments. J Behav Exp Finance.

[38] Choi J, Kim JW, Lee YS (2024). Availability of ChatGPT to provide medical information for patients with kidney cancer. Sci Rep.

[39] Lahat A, Shachar E, Avidan B, Glicksberg B, Klang E (2023). Evaluating the utility of a large language model in answering common patients’ gastrointestinal health-related questions: are we there yet?. Diagnostics (Basel).

[40] Liu S, Wright AP, Patterson BL (2023). Using AI-generated suggestions from ChatGPT to optimize clinical decision support. J Am Med Inform Assoc.

[41] Gordon EB, Towbin AJ, Wingrove P (2024). Enhancing patient communication with Chat-GPT in radiology: evaluating the efficacy and readability of answers to common imaging-related questions. J Am Coll Radiol.

[42] Sallam M, Barakat M, Sallam M (2024). A preliminary checklist (METRICS) to standardize the design and reporting of studies on generative artificial intelligence-based models in health care education and practice: development study involving a literature review. Interact J Med Res.

[43] Bazzari FH, Bazzari AH (2024). Utilizing ChatGPT in telepharmacy. Cureus.

[44] Khlaif ZN, Mousa A, Hattab MK (2023). The potential and concerns of using AI in scientific research: ChatGPT performance evaluation. JMIR Med Educ.

[45] Zhou Y, Moon C, Szatkowski J, Moore D, Stevens J (2024). Evaluating ChatGPT responses in the context of a 53-year-old male with a femoral neck fracture: a qualitative analysis. Eur J Orthop Surg Traumatol.

[46] Bhattacharjee A, Suh J, Ershadi M, Iqbal ST, Wilson AD, Hernandez J (2024). Understanding communication preferences of information workers in engagement with text-based conversational agents. arXiv.

[47] Furini M, Mariani M, Montagna S, Ferretti S (2024). Conversational skills of LLM-based healthcare chatbot for personalized communications. GoodIT ’24: Proceedings of the 2024 International Conference on Information Technology for Social Good.

[48] Janson A (2023). How to leverage anthropomorphism for chatbot service interfaces: the interplay of communication style and personification. Comput Human Behav.

[49] Chaves AP, Gerosa MA (2021). How should my chatbot interact? A survey on social characteristics in human–chatbot interaction design. International Journal of Human–Computer Interaction.

[50] Kiritchenko S, Mohammad S, Barzilay R, Kan MY (2017). Proceedings of the 55th Annual Meeting of the Association for Computational Linguistics (Volume 2: Short Papers).

[51] Thurstone LL (1927). A law of comparative judgment. Psychol Rev.

[52] Christensen RHB (2026). Regression models for ordinal data. Ordinal.

[53] Holm S (1979). A simple sequentially rejective multiple test procedure. Scand J Stat.

[54] Harrell FE, Harrell Jr Frank E (2015). Regression Modeling Strategies With Applications to Linear Models, Logistic and Ordinal Regression, and Survival Analysis.

[55] Agresti A (2010). Analysis of Ordinal Categorical Data.

[56] Abbas Q, Jeong W, Lee SW (2025). Explainable AI in clinical decision support systems: a meta-analysis of methods, applications, and usability challenges. Health Care (Don Mills).

[57] Patel N, Grewal H, Buddhavarapu V, Dhillon G (2025). OpenEvidence: enhancing medical student clinical rotations with AI but with limitations. Cureus.

[58] Brandtzaeg PB, Følstad A, Kompatsiaris I, Cave J, Satsiou A, Carle G, Passani A, Kontopoulos E, Diplaris S, McMillan D (2017). Internet Science.

[59] Laranjo L, Dunn AG, Tong HL (2018). Conversational agents in healthcare: a systematic review. J Am Med Inform Assoc.

[60] Sharma A, Miner A, Atkins D, Althoff T, Webber B, Cohn T, He Y, Liu Y (2020). A computational approach to understanding empathy expressed in text-based mental health support.

[61] Li J, Zhu Z, Zhang R, Lee YC (2025). Exploring the effects of chatbot anthropomorphism and human empathy on human prosocial behavior toward chatbots. Proc ACM Hum-Comput Interact.

[62] Akbulut C, Weidinger L, Manzini A, Gabriel I, Rieser V (2025). All too human? mapping and mitigating the risks from anthropomorphic AI. AIES ’24: Proceedings of the 2024 AAAI/ACM Conference on AI, Ethics, and Society.

[63] Zhang D, Shi Z, Hu H, Han GK (2021). Classification of the use of online health information channels and variation in motivations for channel selection: cross-sectional survey. J Med Internet Res.

[64] Cuadra A, Wang M, Stein LA (2024). The illusion of empathy? notes on displays of emotion in human-computer interaction.

[65] Liu B, Sundar SS (2018). Should machines express sympathy and empathy? Experiments with a health advice chatbot. Cyberpsychol Behav Soc Netw.

[66] Seitz L (2024). Artificial empathy in healthcare chatbots: Does it feel authentic?. Computers in Human Behavior: Artificial Humans.

